# Involvement of Porcine β-Defensin 129 in Sperm Capacitation and Rescue of Poor Sperm in Genital Tract Infection

**DOI:** 10.3390/ijms23169441

**Published:** 2022-08-21

**Authors:** Fanwen Zeng, Mingming Wang, Ju Li, Chengde Li, Xueqing Pan, Li Meng, Li Li, Hengxi Wei, Shouquan Zhang

**Affiliations:** National Engineering Research Center for Breeding Swine Industry, Guangdong Provincial Key Lab of Agro-Animal Genomics and Molecular Breeding, College of Animal Science of South China Agricultural University, Guangzhou 510642, China

**Keywords:** fertilization, porcine β-defensin 129, sperm capacitation, sperm motility, immune

## Abstract

In mammals, β-defensins have been reported to play pivotal roles in sperm protection and fertilization. However, the function and mechanism of porcine β-defensin 129 (pBD129) in the sperm remain unclear. Here, we demonstrate that pBD129 is a glycosylated protein and broadly exists in accessory sex glands and coats the sperm surface. We inhibited the pBD129 protein on the sperm surface with an anti-pBD129 antibody and found that sperm motility was not significantly affected; however, sperm acrosome integrity and tyrosine phosphorylation levels increased significantly with time (*p* < 0.05) during capacitation. These changes were accompanied by an increase in sperm Ca^2+^ influx, resulting in a significantly reduced in vitro fertilization cleavage rate (*p* < 0.05). Further investigation revealed that treatment with recombinant pBD129 markedly restored the sperm motility in semen contaminated with *Escherichia coli*. The results suggest that pBD129 is not only associated with poor sperm motility after genital tract infection but can also protect the spermatozoa from premature capacitation, which may be beneficial for semen preservation.

## 1. Introduction

According to the World Health Organization (WHO), human infertility affects 15% of couples worldwide, and 50% of these cases over the last 30 years have been attributed to male factors [1,2]. Classic causes of male infertility include sperm dysfunction and urogenital tract infections [3]. Sperm motility is one of the most important sperm characteristics, and reduced sperm motility accounts for approximately 18% of male subfertility and infertility cases [4]. Certain associated seminal tract infections can impair sperm vitality and motility [5]. The epididymis plays a critical role in the synthesis and secretion of specific proteins to create a specific luminal fluid microenvironment by synthesizing and secreting different proteins for sperm maturation [6]. In contrast, in the cauda epididymis, various proteins can be secreted to prevent premature sperm activation and capacitation and maintain the quiescence of sperm metabolism to prolong their survival time [7,8,9]. Fully differentiated spermatozoa from epididymal principal cells undergo substantial surface remodeling, which is considered essential for motility and storage during passage through the epididymal duct [10,11].

Defensins are endogenous, small, secretory cationic peptides with six cysteine residues [12,13]. They are classified into α-defensin, β-defensin, and θ-defensin forms in mammals based on their pattern of cysteine spacing and disulfide bond arrangement [14]. Mature β-defensins are expressed in various epithelial cells and are considered important components of the innate immune system with antimicrobial, anti-inflammatory, and pro-inflammatory effects [15,16,17]. Recently, β-defensin has been found to be widely expressed in mammalian male reproductive systems, maintaining a stable environment in the epithelium and the epididymal cavity of the epididymis by playing a bacteriostatic anti-inflammatory role, suggesting that these secretory defensin peptides may contribute to creating distinct microenvironments for sperm maturation and capacitation [18,19]. Previous studies have indicated that human β-defensin 1 (DEFB1) is not only related to sperm bactericidal activity, quality, and egg-penetrating ability, but it interacts with chemokine receptor type 6 (CCR6) in the sperm and also triggers Ca^2+^ mobilization [20]. Homozygous deletion of a cluster of nine β-defensin genes (DefbΔ9) in mice led to alterations in intracellular calcium levels, inappropriate spontaneous acrosome reaction (AR), and profound male infertility [21]. In addition, β-defensin can prevent sperm recognition by the female immune system and may facilitate the delivery of capacitated sperm to the site of fertilization [22]. Although these findings indicate the involvement of a majority of epididymal β-defensins in sperm maturation and fertilization, the possible involvement of other β-defensins in sperm function and fertility has not been reported.

A high expression of *β-defensin 129* (DEFB129) was first reported in porcine epididymis and small intestine in 2006 [23]. Previous studies have found that porcine β-defensin 129 (pBD129) attenuates lipopolysaccharide (LPS)-induced inflammatory responses by decreasing the serum concentrations of inflammatory cytokines [24,25]. In addition, DEFB129 was considered to have high antibacterial and anti-inflammatory activity in the semen of non-obstructive azoospermic men [26]. Comparative transcriptome analysis between the epididymides of yak and cattleyak revealed the downregulation of DEFB129 in cattleyak [27]. A recent study demonstrated that adding antibodies to the fertilization medium for blocking buffalo β-defensin 129 (BBD129) on the sperm surface hinders cleavage, morula, and blastocyst formation rates, thereby hampering fertilization [28]. Although this evidence indicates that DEFB129 secreted by the epididymis has multiple functions, the specific biological functions of the sperm and its fertilization remain unclear. In addition, it has been demonstrated that pigs represent a more relevant anatomical and physiological model that is widely used in biomedical research for humans [29,30]. The present study aimed to test the hypothesis that pBD129 is a novel and critical molecule in the regulation of sperm capacitation and infertility in genital tract infections.

## 2. Materials and Methods

### 2.1. Animals and Samples

Boar tissue and sow ovaries were collected from a local slaughterhouse. Fresh, commercial ejaculated semen was purchased from Guangdong Kaiping Guangsanbao Pig Industry, stored at 17 °C, and transported to the laboratory within 4 h; 6–8 week-old male BALB/C mice were purchased from Guangdong Medical Experimental Animal Center and housed under controlled environmental conditions with free access to water and food, and a 12 h light–dark cycle.

### 2.2. Experimental Design

#### 2.2.1. Experiment 1: Expression and Purification of Recombinant pBD129 Protein, and Preparation of Anti-pBD129 Antibody

For the expression of pBD129 protein, we used the *E. coli* expression system. In addition, we used BALB/c mice to prepare the anti-pBD129 mouse polyclonal antibody and further verified antibody specificity.

#### 2.2.2. Experiment 2: pBD129 in the Sperm and Reproductive System

The experiment was designed to determine whether pBD129 is detectable in the ejaculated sperm and reproductive system of adult landrace boars. Three biological replicates from three different animals for each group were included.

#### 2.2.3. Experiment 3: Effect of pBD129 on Sperm Function

We assessed the effect of pBD129 on sperm motility, AR, and levels of sperm tyrosine phosphorylation during capacitation. Sperm were co-incubated with 1:100 anti-pBD129 antibody at 37 °C for 30 min in mTBM containing BSA and sperm motility was then evaluated using CASA. Sperm tyrosine phosphorylation was evaluated using Western blotting after co-incubating the sperm with 1:100 anti-pBD129 antibody at 37 °C for 60, 120, and 180 min in mTBM containing BSA. The assays were repeated at least three times.

#### 2.2.4. Experiment 4: Effect of pBD129 on Sperm Intracellular Ca^2+^

To assess the effect of pBD129 on sperm intracellular Ca^2+^, sperm were co-incubated with 1:100 anti-pBD129 antibody at 37 °C for 60, 120, and 180 min and evaluated using Fluo-3AM. The assays were repeated at least three times.

#### 2.2.5. Experiment 5: Effect of pBD129 on In Vitro Fertilization

The effect of different anti-pBD129 antibody concentrations in the capacitation and fertilization media on fertilization rate was assessed by diluting the antibody to 1:100, 1:1000, and 1:5000 concentrations for the experiment. The assays were repeated at least three times.

#### 2.2.6. Experiment 6: Effect of Recombinant pBD129 on Sperm Motility in *E. coli*

This experiment was designed to determine whether recombinant pBD129 protein rescued sperm motility in the presence of *E.coli*. For sperm treatment, recombinant pBD129 proteins (final concentration of 0–100 µg/mL) and 2 × 10^6^ CFU/mL of *E. coli* DH5α were co-incubated with sperm at 37 °C for 30 min, and sperm motility was then assessed by CASA. The assays were repeated at least three times.

### 2.3. Preparation of pBD129 Protein and Antibody

Porcine β-defensin 129 cDNA was obtained from porcine epididymis. According to the genomic DNA sequence of *pBD129* (GenBank accession No.NM_001129975.1), we used forward and reverse primers (5′-CGCCATATGATGGAATACTTTGGCTTGGGAAG-3′ and 5′-CCGCTCGAGAGTCATGCTGTCGGCAGGGT-3′, respectively) to amplify the cDNA fragments containing the two indels and then insert the pBD129 into the NdeI/XboI sites of pET28a(+). The recombinant plasmids [pET28a(+)-pBD129] were transformed into *Escherichia* *coli* BL21 (DE3) cells. Single colonies were established in lysogeny broth (LB) medium at 37 °C containing 50 mg/mL kanamycin and cultured accompanied by shaking at 200 rpm/min until the OD_600_ reached 0.6–0.8. Protein expression was induced with 1 mM isopropyl-l-thiogalactopyranoside (IPTG) at 37 °C for 6 h. Cells were harvested using centrifugation at 12,000× *g* for 1 min at 4 °C and lysed by sonication in ice-water bath. After centrifugation at 12,000× *g* for 5 min at 4 °C, the supernatants were loaded onto a Ni-NTA column to obtain purified pBD129 protein according to standard protocol (Ni-NTA QIAexpress Kit manual). The fractions were dialyzed against phosphate buffered saline (PBS) at 4 °C three times for half day each. A BCA protein content detection kit (#KGPBCA, KeyGen Biotech, Nanjing, China) was used for quantitatively analyzing the protein concentrations. Sample purity was evaluated by analyzing sodium dodecyl sulfate-polyacrylamide gel electrophoresis (SDS-PAGE) images and stored at −80 °C until use. The recombinant pBD129 protein was identified using liquid chromatography-tandem mass spectrometry (LC-MS/MS), according to the previously described protocol [31].

Mouse polyclonal antiserum specifically customized for recombinant pBD129 protein was prepared and improved as described previously [32], and the pre-immune sera were also purified as negative control following the same protocol. Briefly, five BALB/c mice were inoculated with 100 μg of recombinant pBD129 every 2 weeks, three times in total. The immune response was confirmed by verifying the binding of serum to the antigen using an enzyme-linked immunosorbent-type assay. The mice were anesthetized, and serum was collected according to animal welfare standards. An affinity-purified polyclonal antibody (immunoglobulin G [IgG] fraction) was prepared using a nickel-NTA column as described previously [33]. The purified antibody was aliquoted in a storage buffer (50% glycerin, 0.1% sodium azide, and 0.1% gelatin) and stored at −80 °C until use.

### 2.4. Antimicrobial and Hemolytic Activity Assays

The minimum inhibitory concentration (MIC) of the recombinant pBD129 protein was determined as previously described [34]. *Streptococcus dysgalactiae* ATCC12394 and *E. coli* DH5α were used to measure the antibacterial activity. Briefly, recombinant pBD129 protein was continuously doubling diluted from 200 μg/mL, and 100 μL/well was added to 96-well microplates containing a 100 μL/well concentration of 1 × 10^5^ colony-forming unit (CFU)/mL bacteria. Phosphate buffer saline (PBS) of the same volume was used as negative control. Finally, the 96-well microplate was incubated at 37 °C for 24 h, and bacterial growth was measured at OD_600_ nm using a spectrophotometer.

In addition, the sperm antibacterial activity against *E. coli* was determined using the CFU assay, as previously described [35]. Briefly, bacteria were diluted to 2 × 10^6^ CFU/mL and incubated with sperm overnight at 37 °C with anti-pBD129 antibody. Post measurement at OD_600_ nm, the resultant colonies were spread on LB plates and incubated overnight at 37 °C to obtain a countable number. PBS of the same volume was used as negative control.

Hemolytic activity was measured as previously described [24]. Briefly, fresh porcine blood was washed three times, centrifuged at 1500× *g* for 10 min at room temperature with PBS, resuspended in PBS to a final dilution of 4% (*v*/*v*), and added to a 96-well microplate. Subsequently, recombinant pBD129 protein was serially diluted from 200 μg/mL by a factor of 2; 150 μL aliquots were then added to 96-well microplates and incubated at 37 °C for 1 h. After incubation, the plate was centrifuged for 5 min at 1500× *g*, and the absorbance of 150 μL supernatant from each well was measured at 450 nm using a spectrophotometer. Equal volumes of PBS and 0.1% Triton X-100 were used as negative and positive controls, respectively.

### 2.5. De-N-Glycosylation of Recombinant pBD129 and Specificity Analysis of Antibodies

Human HEK293T cells were seeded in 96-well plates at 1 × 10^5^ cells/mL or in 6-well plates at 2 × 10^6^ cells/mL and cultured in DMEM with 10% FBS in 5% CO_2_ at 37 °C in humidified air until the cell confluence reached 50%. The *pBD129* gene (GenBank accession no. NM_001129975.1) was synthesized and cloned into BamHI/XhoI sites of pcDNA3.1(+) (Sangon Biotech Co., Ltd., Guangzhou, China). The pcDNA3.1-pBD129 and pcDNA3.1 plasmids were transfected into HEK293T cells using Lipofectamine 3000 (Invitrogen). HEK293T cells transfected with an empty pcDNA3.1 plasmid served as the negative control. The supernatant of the treated HEK293T cells was collected after transfection for 40 h and used for analyzing the N-glycosidic bonds of glycoproteins and the specificity analysis of anti-pBD129 polyclonal antibody. For glycoprotein analysis, the supernatant (25 μg) was incubated with 10 mU glycopeptidase F (#4450; Takara, Dalian, China) for 20 h at 37 °C in 2.5 μL native buffer and then analyzed using western blotting.

### 2.6. Western Blotting

The samples were replaced with lysis buffer (#KGP250; KeyGen Biotech) containing phosphatase and protease inhibitors and phenylmethylsulfonyl fluoride. After homogenization, the samples were sedimented at 12,000× *g* for 5 min at 4 °C. The supernatant was quantified using the BCA protein detection kit.

For Western blot analysis, samples were separated using 10% SDS-PAGE and transferred onto a polyvinylidene difluoride (PVDF) membrane (Millipore, Burlington, MA, USA). PVDF membranes were blocked with 5% milk powder in Tris-buffered saline with 0.1% Tween (TBST) for 1 h at room temperature, followed by incubation with anti-pBD129 primary antibody diluted 1:1000 and anti-tubulin primary antibody (#AF0001, Beytime Biotech, Shanghai, China) diluted 1:1000 overnight at 4 °C and then washed three times with TBST before incubation with horseradish peroxidase (HRP)-conjugated goat anti-rabbit IgG secondary antibody or HRP-conjugated goat anti-mouse IgG secondary antibody diluted 1:3000 at room temperature for 1 h. Negative control experiments were performed using normal mouse IgG as the primary antibody. After washing three times with TBST, the membranes were visualized using the ECL plus Western blotting detection system (Thermo Fisher Scientific, Waltham, MA, USA) and an imaging system (Tanon 5200). To assess relative protein levels, the experiment was repeated at least three times and the average protein intensities were quantified using Image J software (NIH, Bethesda, Rockville, MD, USA) and normalized to tubulin levels.

### 2.7. Immunohistochemistry

Cryosections of pBD129 in adult boar epididymis tissues were excised and fixed in fresh 4% paraformaldehyde in PBS at pH 7.4 overnight at 4 °C, washed four times in PBS, and cryopreserved for 30 h in 30% sucrose in PBS accompanied by stirring at 4 °C; the samples were flash-frozen on dry ice and stored at −80 °C and then analyzed on 25 μm tissue sections mounted on siliconized slides at −25 °C. Sections were blocked with 10% goat serum in PBS for 1 h and incubated with a primary anti-pBD129 antibody (diluted 1:100 in blocking solution) overnight at 4 °C. The primary antibody replaced with normally diluted mouse IgG was used as negative control. After washing three times, the sections were incubated with HRP-conjugated secondary antibodies at a final dilution of 1:400 for 1 h at 37 °C. Staining was visualized using a 3,3′-diaminobenzidine (DAB) Kit and slides were counterstained with hematoxylin. Sections were observed using an Olympus BX53F microscope, and the mean density of positive immunostaining was analyzed using a commercial image analysis software.

### 2.8. Indirect Immunofluorescence

For immunofluorescence staining, semen containing 5 × 10^6^ sperm/mL was washed three times with PBS by centrifugation at 2500× *g* for 5 min. After coating them on coverslips and drying for 50 min at room temperature, the sperm samples were fixed with 4% paraformaldehyde (*w/v*) for 1 h, followed by washing three times in PBS. The coverslips were blocked with 10% goat serum at room temperature for 1 h and incubated with a primary polyclonal anti-pBD129 antibody. After three further washes with PBS, the samples were incubated with Alexa Fluor 568-goat anti-mouse IgG (dilution 1:100) at 37 °C for 1 h, followed by incubation with Hoechst 33342 diluted to 1:100 (#H3570; Life Technologies, Carlsbad, CA, USA) for 10 min at room temperature in the dark. Finally, the slides were washed three times in PBS and analyzed using a fluorescence microscope (BX53F, Olympus, Tokyo, Japan). Negative control experiments were performed using normal mouse IgG as the primary antibody.

### 2.9. Sperm Motility Assay

Semen samples purchased from a commercial company were stored at 17 °C for 4 h and transferred to our laboratory for evaluating the sperm parameters including motility, morphology, and membrane integrity. After washing three times in Dulbecco’s phosphate-buffered saline (DPBS) containing 1 mg/mL bovine serum albumin (BSA), the sperm were resuspended to 2–5 × 10^6^ sperms/mL. Sperm motility was evaluated using a computer-assisted sperm analysis (CASA; Integrated Sperm Analysis System, ISAS, V1.0; Proiser, Valencia, Spain) system equipped with a warm stage. Subsequently, 5 μL drop per sample was placed in 20 μm deep disposable counting chambers (Leja, NieuwVennep, the Netherlands) and analyzed using the CASA system. The evaluated individual kinematic parameters included total motility (TM, %), progressive motility (PM, %), curvilinear velocity (VCL, μm·s^−1^), straight-line velocity (VSL, μm·s^−1^), average path velocity (VAP, μm·s^−1^), amplitude of lateral head displacement (ALH, μm), beat cross frequency (BCF, Hz), wobble coefficient (WOB, %), linearity (LIN, %), mean angular displacement (MAD, degree), and straightness (STR, %).

### 2.10. Evaluation of Sperm AR

Sperm AR was evaluated using 1% FITC-conjugated peanut agglutinin from *Arachis hypogaea* (10 μg/mL FITC-PNA, #L7381, Sigma, Marlborough, MA, USA) as previously described [36]. Briefly, sperm capacitation was induced using modified Tris-buffered medium (mTBM; 11.3 nM NaCl, 0.3 mM KCl, 1 mM CaCl_2_, 0.5 mM pyruvate, 1.1 mM glucose, 2 mM TRIS) containing 1 mg/mL BSA and anti-pBD129 antibody (1:100) by exposing the sperm to 5% CO_2_ at 37 °C for 180 min. Mouse IgG served as negative control. The sperm were then treated with progesterone for 60 min. After incubation with FITC-PNA at room temperature for 30 min, the samples were smeared onto glass slides and analyzed using fluorescence microscopy (BX53F, Olympus, Tokyo, Japan). For each sample, approximately 400 sperms were scored to classify the different patterns into reacted (with fluorescence in the acrosomal region) or non-reacted (without fluorescence in the acrosomal region).

### 2.11. Measurement of Sperm Intracellular Ca^2+^

Ca^2+^ levels in the spermatozoa were examined by applying the fluorescent probe, Fluo3-AM (#46393, Sigma) and using single-sperm intracellular Ca^2+^ imaging, as previously described [37] with minor modifications. Briefly, each group was incubated with anti-pBD129 antibody (1:100) and loaded with Fluo3-AM (5 μM) at 37 °C for 30 min in the dark in mTBM without BSA, and subsequently washed in mTBM with centrifugation at 500× *g* for 5 min to remove any free Fluo3-AM. Sperm concentration was adjusted to 5 × 10^6^ sperm/mL using a blood cell meter, and the sperm were incubated at room temperature for 20 min for complete hydrolyzation of acetoxymethyl with cytoplasmic esterase. Mouse IgG was used as a negative control. The results were analyzed using an inverted microscope (IX-71, Olympus) at excitation and emission wavelengths of 488 nm and 525 nm, respectively. The mean fluorescence intensity of individual spermatozoa was quantified with at least three repetitions.

### 2.12. In Vitro Fertilization (IVF) Assay

Cell culture and IVF were performed as previously described [38]. Briefly, porcine ovaries were collected from a local abattoir and transported to the laboratory at 30–35 °C. Cumulus–oocyte complexes (COCs) were aspirated from 3–6 mm diameter follicles and rinsed three times in DPBS containing 0.1% (*w/v*) polyvinyl alcohol. Subsequently, the COCs were transferred to 500 μL in vitro maturation medium (Medium 199 supplemented with 10 IU/mL pregnant mare serum gonadotropin, 10 IU/mL human chorionic gonadotropin, 0.1 mg/mL L-cysteine, 10% porcine follicular fluid, 10% fetal bovine serum, and 10 ng/mL epidermal growth factor) covered with mineral oil. Oocytes were then matured for 40–44 h at 38.5 °C in 5% CO_2_. After cumulus removal using hyaluronidase, the oocytes were washed three times in the IVF medium and transferred into 50 μL droplets of the medium covered with mineral oil. Spermatozoa were capacitated with mTBM containing BSA to a concentration of 2 × 10^6^ sperm/mL. After co-incubation with sperm for 6 h, the oocytes were transferred to 500 μL PZM-3 medium (Medium 199 supplemented with 0.6312 g/mL NaCl, 0.2106 g/mL NaHCO_3_, 0.0746 g/mL, 0.0048 g/mL KH_2_PO_4_, 0.0098 g/mL MgSO_4_·7H_2_O, 0.0436 g/mL Ca-(Lactate)·5H_2_O, 0.0022 g/mL Na-pyruvate, 0.0146 g/mL L-glutamine, 0.0545 g/mL hypotaurine, and 0.3 g/mL BSA) covered with mineral oil and cultured at 38.5 °C for 48 h in 5% CO_2_. For antibody treatment, boar sperm were incubated with anti-pBD129 antibody, and mouse IgG was used as negative control. Fertilization rates were evaluated under a microscope at 200 × magnification (Leica Microsystems; Wetzlar, Germany).

### 2.13. Statistical Analysis

All statistical analyses were performed using SPSS software (version 21.0; IBM Corp., Armonk, New York, NY, USA) and GraphPad Prism 9 software (version 9.0.0, La Jolla, CA, USA). Data are presented as mean ± standard error of the mean. Statistical differences among groups were determined using the Student’s *t* test, chi-square test, and ANOVA. All *p* values  <  0.05 were considered statistically significant.

## 3. Results

### 3.1. Expression, Purification, and Characterization of Recombinant pBD129 Protein

Obtaining an active pBD129 peptide is an important step in addressing the roles of pBD129 in immunity and fertility. The pBD129 protein was expressed in *E. coli* BL21 (DE3) and purified using a Ni-NTA agarose column. The molecular weight of the pure recombinant pBD129 protein was similar to that of the predicted recombinant protein (21 kDa), indicating the successful expression of recombinant protein (Supplementary Materials Figure S1A). To further evaluate the recombinant pBD129 protein, the purified peptide was identified using mass spectrometry (LC-MS/MS). The amino acid sequence of pBD129 obtained from the LC-MS/MS data was matched with that present in the NCBI database (Accession No. NP_001123447.1), and the result revealed greater than 70% match with NP_001123447, which was based on our previous design (Supplementary Materials Figure S1B).

**Figure 1:**
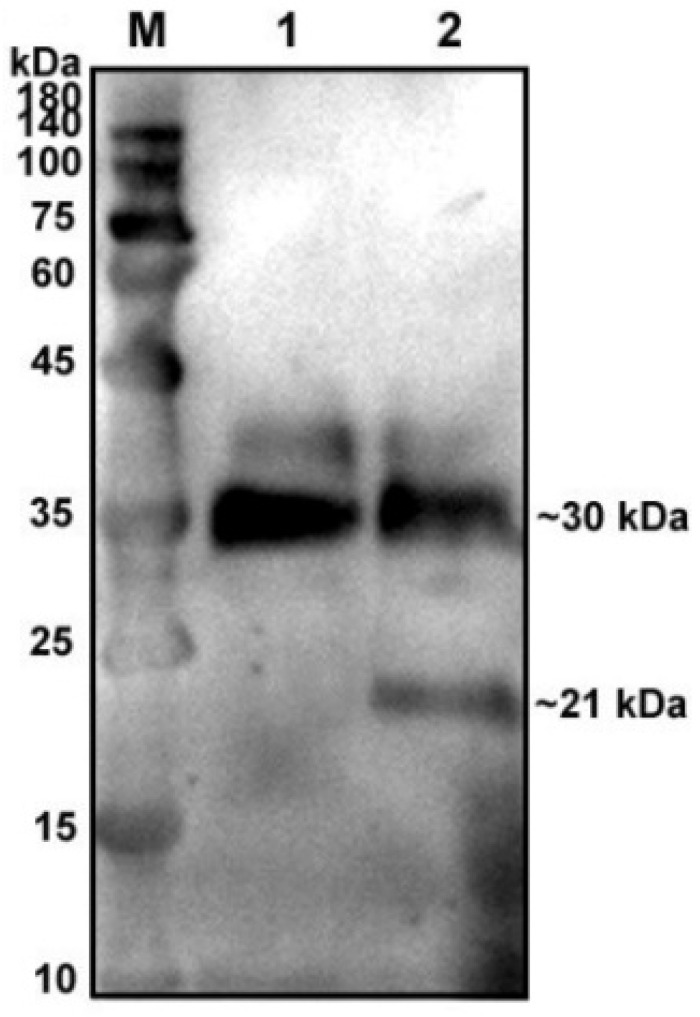
Glycosylation analysis of recombinant pBD129 protein in HEK293T cells. M: 180 kDa protein marker. Lane 1: supernatant of HEK293T cells transfected with pcDNA3.1(+)-pBD129 without glycosidase F treatment (Only a 30 kDa band). Lane 2: The supernatant of HEK293T cells transfected with pcDNA3.1(+)-pBD129 was treated with glycosidase F (two bands at 30 kDa and 21 kDa).

To verify glycosylation modification in the native protein of pBD129, we treated the supernatant of pBD129-transfected cells with glycosidase F at 37 °C for 20 h and found a single target band at 21 kDa (Figure 1) compared with that in the control group, indicating that the native protein pBD129 was glycoprotein-modified, resulting in increased Western blot bands.

**Figure 2:**
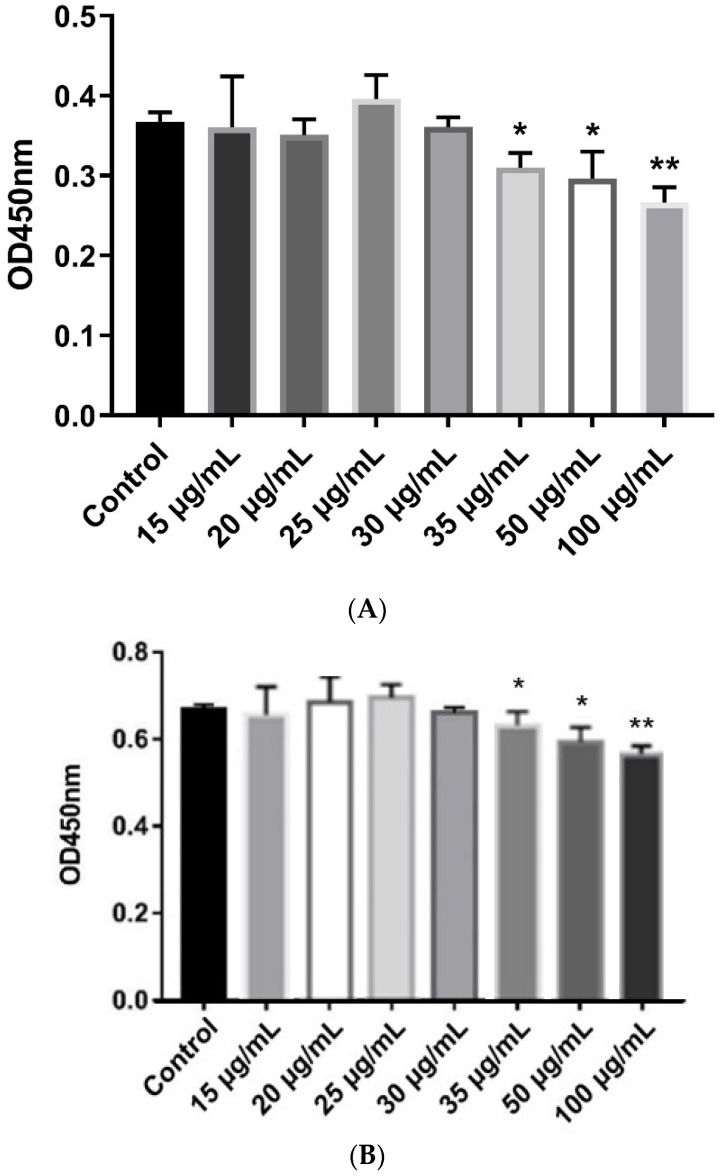
The minimum inhibitory concentration (MIC) assays of recombinant pBD129 protein of *E. coli* expression system. (**A**) The MIC of recombinant pBD129 protein against *E. coli* DH5α was 35 μg/mL. (**B**) The MIC of recombinant pPBD129 protein against *Streptococcus dysgalactiae* was 35 μg/mL. Data are presented as the mean± SEM, *n* = 3. * *p* < 0.05, ** *p* < 0.01.

**Figure 3:**
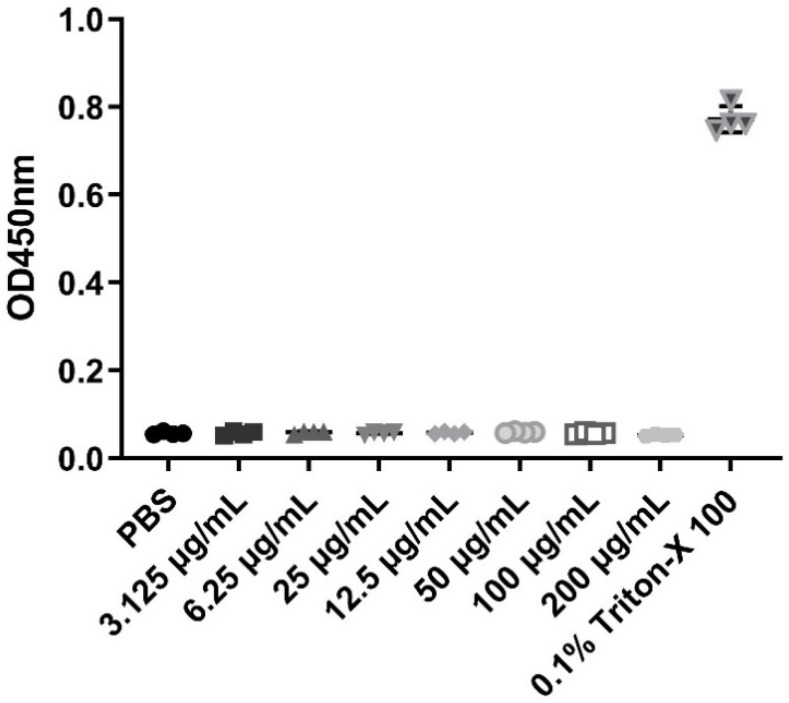
The hemolytic activity of recombinant pBD129 protein of *E. coli* expression system. The X-axis coordinates corresponding to the symbols in the figure are the respective groups. The same volume PBS and 0.1% Triton X-100 were used as negative controls and positive controls, respectively. *n* = 3.

β-defensins generally display a broad range of antibacterial activities. In this study, MIC assays were used to evaluate the antibacterial activities of the recombinant pBD129 protein against *E. coli DH5α* and *Streptococcus dysgalactiae ATCC12394* [25]. As illustrated in Figure 2, the recombinant pBD129 protein exhibits dose-dependent antimicrobial activity against *E. coli DH5α* and *S. dysgalactiae*, and MICs of 35 μg/mL for both vectors. The hemolytic activity of the recombinant pBD129 protein was determined using complete pig blood, with 1 × PBS as a 0% negative control and 0.1% Triton-X 100 as a 100% positive control. We found that recombinant pBD129 protein treatment at doses ranging from 0 to 256 μg/mL had no detrimental effect on the blood cells, while 0.1% Triton-X 100 produced hemolysis (Figure 3).

### 3.2. Localization of pBD129 in Sperm and Reproductive System

After affinity purification, the concentration of pBD129 polyclonal antibody was measured using a protein content detection kit (#KGPBCA, KeyGen Biotech), and the purified antibody was adjusted to 1 mg/mL. Subsequently, the supernatant of HEK293T cells transfected with pcDNA3.1-pBD129 plasmid was assessed using Western blot, which revealed that the specificity of pBD129 polyclonal antibody was not cross-reactive, and the supernatant was therefore used for further experiments (Figure S2).

**Figure 4:**
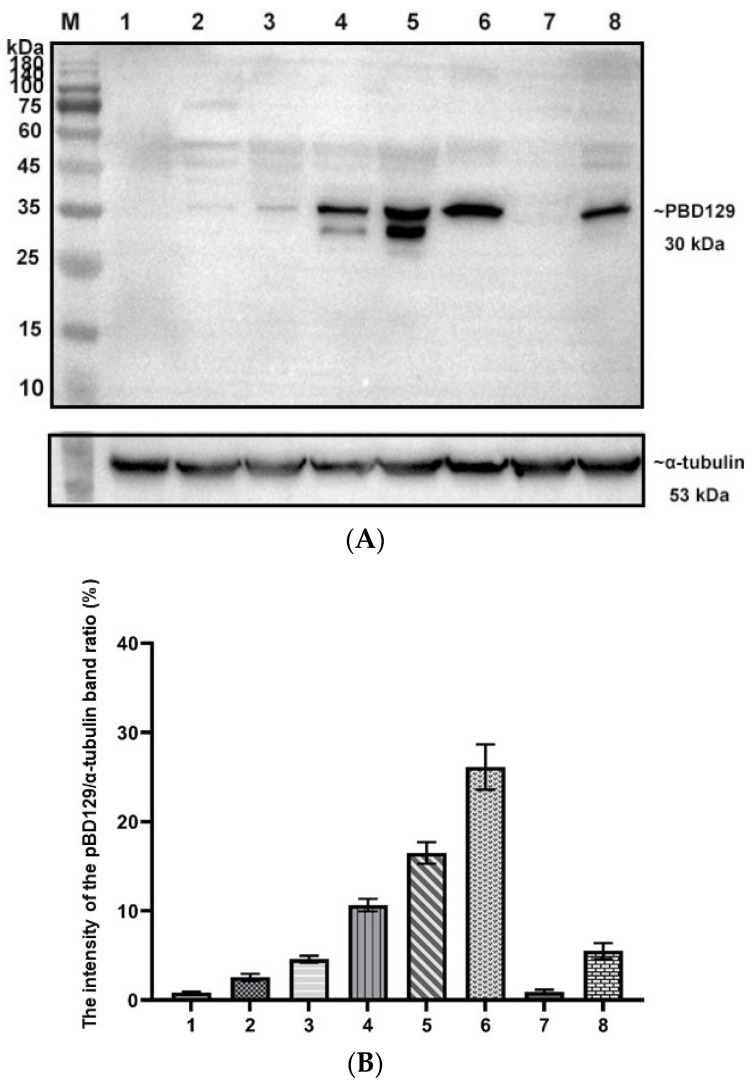
Expression and distribution of pBD129 in the porcine reproductive system. (**A**) Western blot analysis of pBD129 protein in reproductive organs of adult boars. M: 180 kDa protein marker; Lane 1: Testicles; Lane 2: Caput epididymis; Lane 3. Corpora epididymis; Lane 4: Caudal epididymis; Lane 5: Vas deferens; Lane 6: Seminal vesicle gland; Lane 7: Bulbous urethral gland; Lane 8: Prostate. (**B**) The intensity of the pBD129 protein band of Western blot analysis was compared by ImageJ software. α-tubulin as internal control and normalization. Data are presented as the mean ± SEM, *n* = 3.

**Figure 5:**
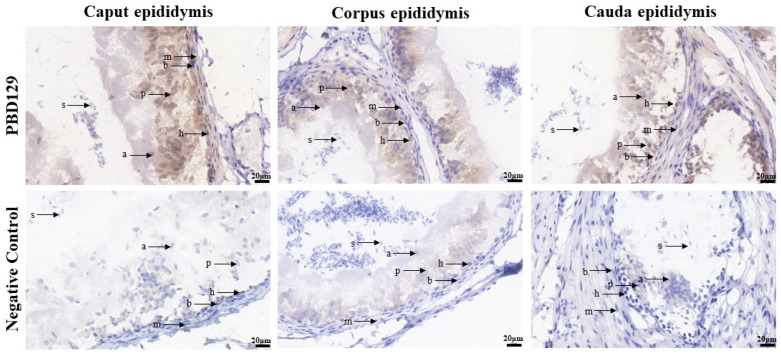
Immunohistochemistry analysis of pBD129 protein localization in the epididymis from adult boar. a, apical cells; b, basal cells; h, halo cells; m, myoid cells; p, principal cells; s, sperm.

**Figure 6:**
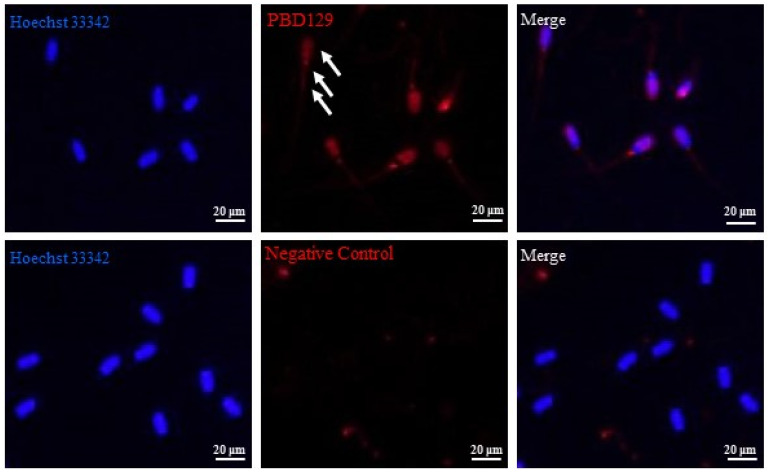
Indirect immunofluorescence analysis of the localization of pBD129 protein in sperm. White arrows indicate pBD129 protein abundance. Hoechst33342 indicates nuclear staining; red indicates pBD129 protein abundance; Merge is nuclear staining and pBD129 protein overlay.

PBD129 protein expression in the reproductive system of adult landrace boars was analyzed using Western blotting. As presented in Figure 4, pBD129 was expressed in the caput, corpora, and caudal epididymides, vas deferens, seminal vesicles, and prostate but not in the testis and bulbourethral gland. Immunolocalization analysis was performed to further confirm the presence of pBD129 in specific epididymal cell types and subcellular compartments. PBD129 exhibited concentrated expression in the luminal aspect of the epithelium in the caput, corpus and caudal regions of the epididymis, compared with that in the negative control groups (Figure 5). The pBD129 protein in the spermatozoa was then localized using immunofluorescence, which revealed the presence of pBD129 along the entire periphery of the boar spermatozoa; the negative controls did not fluoresce upon excitation (Figure 6).

### 3.3. Blocking pBD129 on Sperm Surface Promotes AR and Tyrosine Phosphorylation but Hinders Cleavage

**Figure 7:**
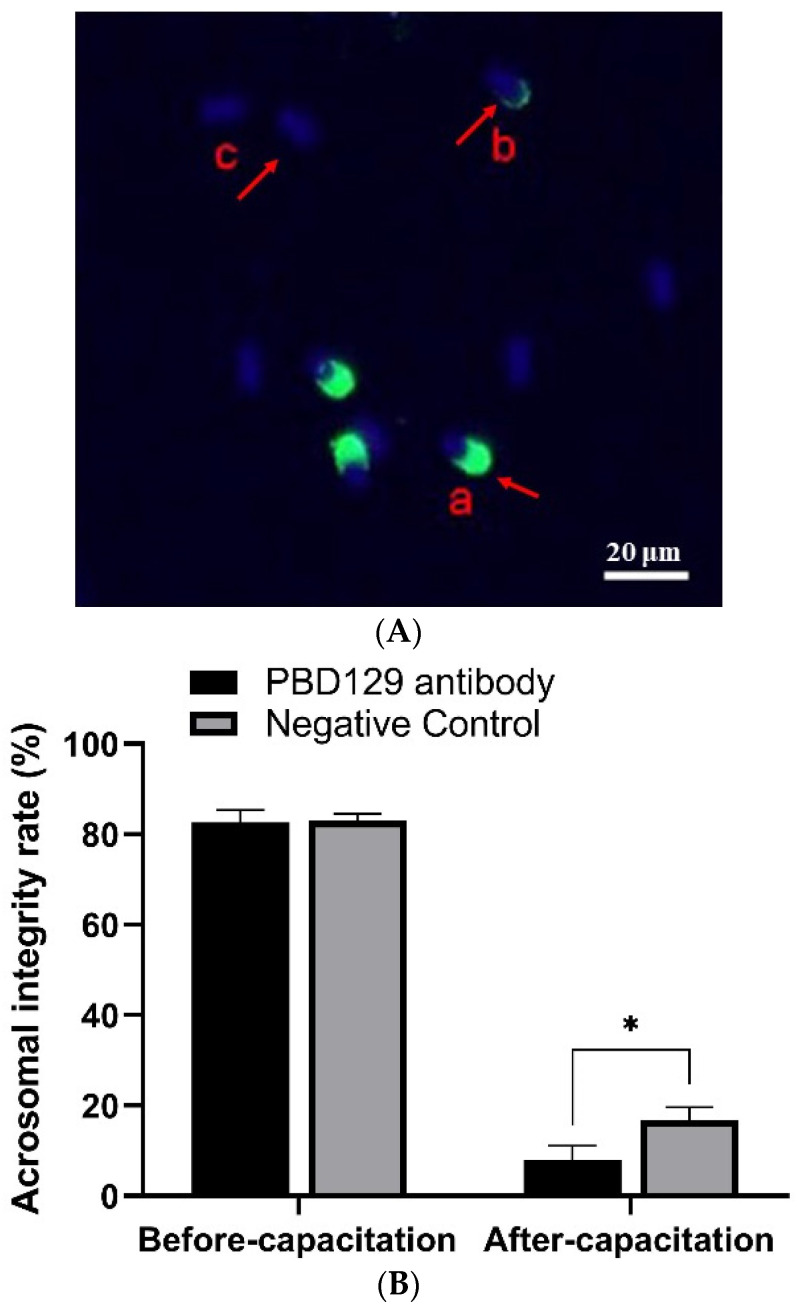
Effects of anti-pBD129 antibody on the rate of acrosome reaction after sperm capacitation in vitro. (**A**) Sperm in different statuses. Sperm samples were fixed with FITC-PNA and Hoechst33342, and scored according to the state of acrosome. a: sperm has not experienced acrosome reaction, and b and c indicate that acrosome reaction has occurred. (**B**) Analysis of sperm acrosome reaction rate before and after capacitation with anti-pBD129 antibody. The mouse IgG as negative control. Data are presented as the mean ± SEM, *n* = 3. * *p* < 0.05.

**Figure 8:**
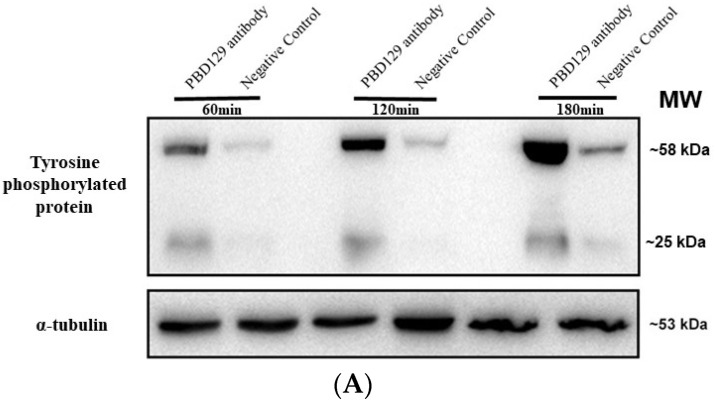

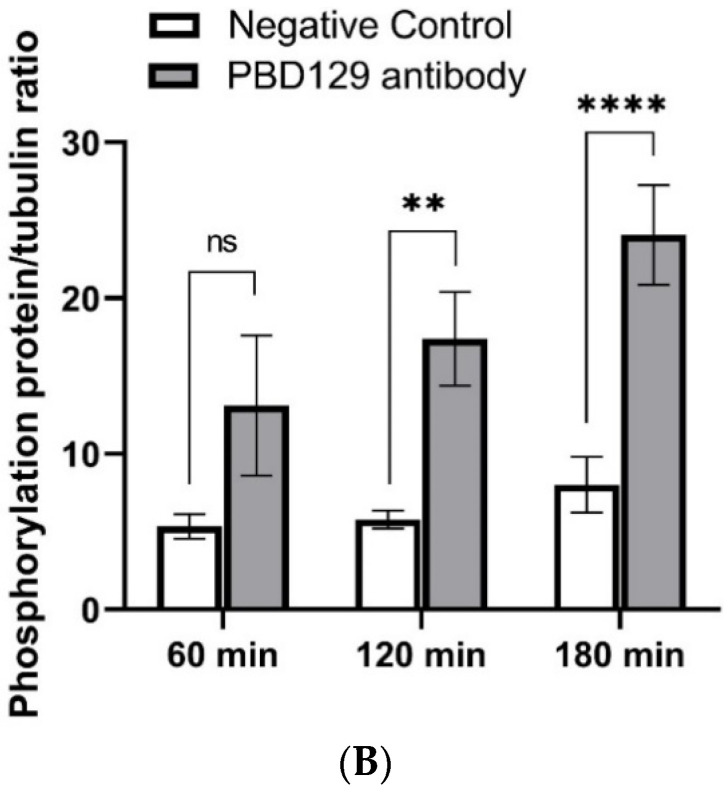
Effects of anti-pBD129 antibody on total protein tyrosine phosphorylation after sperm capacitation in vitro. (**A**) Western blot analysis of total protein tyrosine phosphorylation levels of in vitro capacitated sperm treated with anti-pBD129 antibody and negative control. (**B**) Tyrosine phosphorylation level was analyzed by Image J for gray value analysis, and α-tubulin was normalized. Negative control was mouse IgG. Data are presented as the mean± SEM, *n* = 3. ** *p* < 0.01; **** *p* < 0.0001.

**Figure 9:**
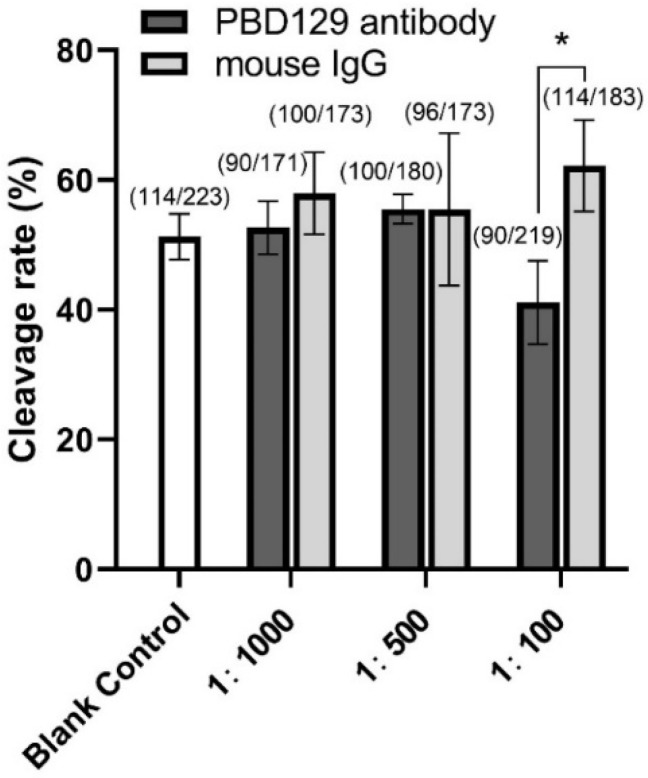
Effects of anti-pBD129 antibody treatment on sperm cleavage rate in in vitro fertilization (IVF). Sperm were co-incubated with sperm 1:100, 1:500, and 1:1000 diluted anti-pBD129 antibody for 30 min, and a relative concentration of anti-pBD129 antibody was added to the fertilization fluid. The corresponding concentrations of mouse IgG were co-incubated with sperm as a negative control. Blank control was untreated. The total number of oocytes used in each group were noted above the column. Data are presented as the mean± SEM, *n* = 3. * *p* < 0.05.

The sperm was treated with anti-pBD129 antibody to confirm the role of pBD129 in the reproductive performance of boars. Compared with the samples in the negative control group, treating the spermatozoa for 30 min failed to affect their motility (Table 1). However, as the Figure 7 and Figure 8 shown that the treatment significantly increased the number of ARs and the level of tyrosine phosphorylation at 120 and 180 min owing to sperm capacitation (*p* < 0.05). In addition, as illustrated in Figure 9, the antibody diluted to 1:100 significantly reduced the cleavage rate in the sperm (*p* < 0.05).

**Figure 10:**
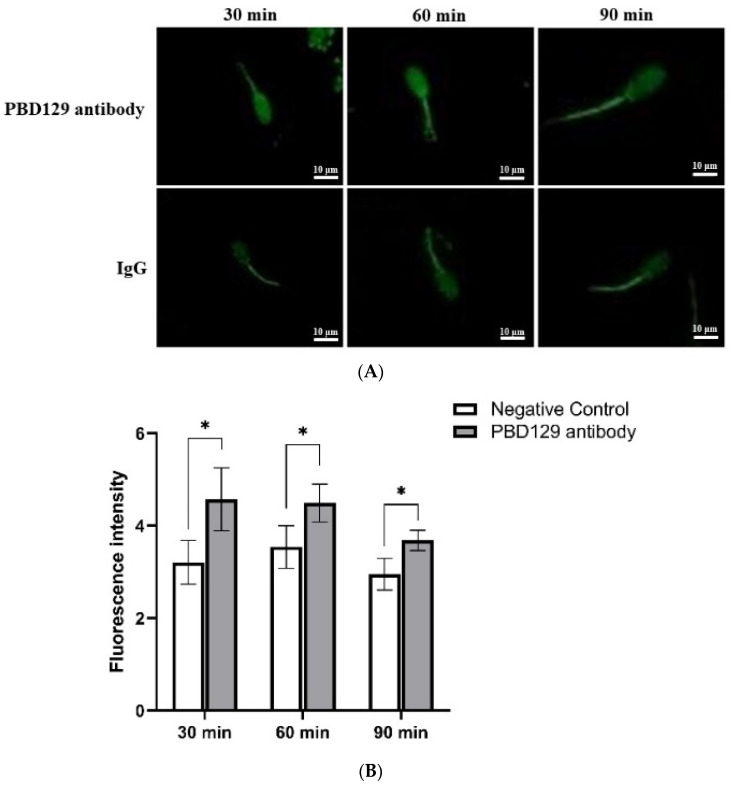
The effect of anti-pBD129 antibody on the influx of Ca^2+^ after sperm capacitation in vitro. (**A**) The influx of Ca^2+^ is presented as the mean fluorescence intensity of Fluo-3. (**B**) Statistical analysis of mean fluorescence intensity values. The mouse IgG as negative control. Data are presented as the mean± SEM, *n* = 3. * *p* < 0.05.

The concentration of Ca^2+^ in the sperm can affect sperm capacitation. As the capacitation process is Ca^2+^-dependent, we used single-sperm Ca^2+^ imaging analysis to determine whether pBD129 affects sperm Ca^2+^ influx. The mean fluorescence intensity of Fluo-3AM (indicating intra-sperm Ca^2+^) in the spermatozoa increased significantly at 30, 60, and 90 min in samples treated with anti-pBD129 antibody compared with those in the negative control (Figure 10).

### 3.4. PBD129 Protein Contributes to Motility and Antibactericidal Activity in the Sperm

Sperms from individuals with genital tract infections or those stored at 17 °C are frequently attacked by bacteria. Therefore, we confirmed that *E. coli* DH5α infection in spermatozoa significantly reduced sperm motility (Supplementary Materials Table S1). To evaluate whether recombinant pBD129 protein can alleviate sperm motility after bacterial contamination, we treated contaminated sperms with recombinant pBD129 protein at a final concentration of 6.25–100 μg/mL. The results revealed that sperm motility parameters increased significantly at 6.25–25 μg/mL recombinant pBD129 protein concentration, but decreased significantly at 50 μg/mL and 100 μg/mL concentrations when compared with the negative control (Table 2). We speculate that high concentrations of recombinant pBD129 protein are harmful or non-beneficial to the sperm.

To further verify whether the pBD129 protein on the sperm surface also performs a bacteriostatic function, we blocked pBD129 protein on sperm surface with anti-pBD129 antibody and then examined its effect on bactericidal activity, with mouse IgG as the control antibody. As shown in Table 3, the addition of anti-pBD129 antibody significantly increased the bacterial concentration (0.748 ± 0.060), whereas that of mouse IgG did not, suggesting that the pBD129 protein performs an antibacterial activity in sperm (Supplementary Materials Figure S3).

## 4. Discussion

Infertility is a major challenge in clinical reproductive medicine and affects 8–12% of couples worldwide, with male factors being a major or contributing cause in approximately 50% of couples [39]. Patients with epididymitis have marked changes in sperm protein composition, which may be one of the factors contributing to male infertility after epididymitis episodes. Accumulating evidence suggests that β-defensins play a broad role in regulating early innate immune responses and inducing adaptive immunity. However, the molecular pathogenesis of most infertility conditions is poorly understood. In this study, we demonstrated the dual role of pBD129 in protecting boar fertility.

The sperm maturation process and the ability to gain capacitation involve modification of the sperm surface by different proteins secreted in various specialized regions of the epididymal epithelium. β-Defensins protect sperm from premature capacitation. The homozygous deletion of nine β-defensin genes (DEFBΔ9) in mice results in male sterility, with reduced sperm motility in mutants that exhibit precocious sperm acquisition compared with the wild type. Although the ability to react with spontaneous acrosomes increased, the ability to bind to the zona pellucida of oocytes decreased, and the calcium content of the sperm increased significantly [21]. In another study, rats deficient in DEFB23/26 or DEFB23/26/42 were found to be infertile, with reduced sperm motility, precocious capacitation, increased spontaneous AR, and significantly increased intra-sperm Ca^2+^ [40]. The sperm motility of the anti-pBD129 antibody did not decrease or increase after incubation with capacitated sperms. Sperm motility after capacitation may have reached the final stage, and inhibition of the pBD129 protein did not increase it. However, we also observed that anti-pBD129 antibody inhibition significantly increased intracellular Ca^2+^, and inhibition of pBD129 protein directly triggered calcium mobilization in normal sperm, suggesting a major role of pBD129 in promoting sperm motility. It should also be noted that inhibition of pBD129 protein may increase the post-capacitation AR by progesterone and increase the protein tyrosine phosphorylation levels, suggesting that it may play an important role in preventing premature sperm capacitation.

Asthenozoospermia is commonly associated with epididymitis caused by *E.* *coli*, resulting in reduced sperm motility and bactericidal activity. Multiple β-defensin isoforms synergistically promote innate immunity in the genitourinary system, exhibit bactericidal activity, and promote sperm motility [41]. *E. Coli*-induced infertility is attributable to reduced sperm motility or increased oxidative stress from infection, both of which may result from a deficiency of β-defensins in the sperm. Previous studies have found that Bin1b, which is expressed in the rat epididymis, plays a role in male fertility by promoting the motility of immature sperm and strengthening host defenses in the reproductive tract [42]. A recent study demonstrated that DEFB114 protein exhibits broad-spectrum antibacterial activity and can also rescue LPS-induced reduction in human sperm motility in vitro, suggesting that the rescue effect of human DEFB114 on sperm motility may be secondary to its primary bacteriostatic effect [43]. In this study, in vitro experiments demonstrated that adding *E. coli* to the semen caused a decrease in sperm motility and co-incubation with recombinant pBD129 protein alleviated sperm damage. The results revealed that spermatozoa were relieved at recombinant pBD129 protein concentrations below 100 μg/mL, and the best sperm motility was observed at concentrations of 6.25–12.5 μg/mL recombinant pBD129 protein. In addition, after incubating capacitated sperm with anti-pBD129 antibody, sperm motility did not decrease significantly; however, we did not incubate the obtained sperm from the boar epididymal head with recombinant pBD129 protein to observe the effect on sperm maturation. We hope that future experiments can be performed to verify the effect of pBD129 on sperm maturation. In vitro co-culture of β-defensin antibodies with sperm results in a significant decrease in sperm motility, resulting in decreased fertility and embryonic developmental failure, possibly because of the diminished sperm ability to acquire forward motility and fertilization during passage through the epididymis [44]. Our results suggest that pBD129 protein forms an antibacterial barrier around boar sperm, effectively protecting the semen from microbial contamination and promoting improved fertilization rates.

## 5. Conclusions

We found that pBD129 is glycosylated. The pBD129 protein covers the entire sperm surface, and inhibiting the activity of pBD129 protein increases the sperm acrosome integrity rate and protein tyrosine phosphorylation level while reducing the embryo cleavage rate, which may be caused by excessive Ca^2+^ influx in the sperm. It was also found that the pBD129 protein protected the sperm from damage caused by *E. coli*, thereby rescuing sperm motility. The above research results reveal the expression regulation mechanism and biological function of pBD129, provide new ideas for improving sperm fertility and reproductive performance, and present clues to improve the molecular diagnosis and treatment of male infertility.

## Figures and Tables

**Table 1:** Sperm motility parameter correlation analyses of negative control and anti-pBD129 antibody-treated spermatozoa.

Motility Parameters	Negative Control	Anti-pBD129 Antibody
TM (%)	83.30 ± 2.51 ^A^	80.07 ± 3.89 ^A^
PM (%)	75.43 ± 5.24 ^A^	77.17 ± 2.87 ^A^
VSL (μm/s)	52.70 ± 1.21 ^A^	52.53 ± 2.45 ^A^
VCL(μm/s)	37.17 ± 1.87 ^A^	36.63 ± 0.98 ^A^
VAP (μm/s)	76.29 ± 3.21 ^A^	75.90 ± 2.58 ^A^
ALH (μm)	20.89 ± 1.67 ^A^	21.73 ± 0.53 ^A^
WOB (%)	87.5 ± 0.5 ^A^	80.5 ± 5.5 ^A^
BCF (Hz)	0.78 ± 0 ^A^	0.82 ± 0.015 ^A^
LIN (%)	61 ± 1 ^A^	61.5 ± 0.5 ^A^
MAD (°)	85.09 ± 16.48 ^A^	68.77 ± 4.56 ^A^
STR (%)	86 ± 1 ^A^	87 ± 0 ^A^

TM, total motility; PM, progressive motility; VCL, curvilinear velocity; VSL, straight line velocity; VAP, average path velocity; ALH, mean amplitude of head lateral displacement; WOB, wobble; BCF, beat cross frequency; LIN, linearity; MAD, mean angular displacement; STR, straightness; Values are expressed as mean ± standard error of the mean; saliency analysis using the Student’s *t* test. Different labeled letters represent significant differences (*n* = 3, *p* < 0.05).

**Table 2:** Effects of recombinant pBD129 protein of *E. coli* expression system on sperm motility under *E. coli* DH5α contamination.

Parameters	PBS	6.25 μg/mL	12.5 μg/mL	25 μg/mL	50 μg/mL	100 μg/mL
TM(%)	69.05 ± 4.28 ^A^	76.49 ± 3.74 ^A^	85.18 ± 2.35 ^B^	79.18 ± 2.93 ^B^	68.31 ± 2.93 ^A^	58.76 ± 4.78 ^B^
PM (%)	34.76 ± 2.84 ^A^	49.3 ± 7.28 ^B^	56.90 ± 4.28 ^B^	46.48 ± 2.74 ^B^	37.60 ± 2.40 ^A^	36.26 ± 6.68 ^A^
VSL (um/s)	17.41 ± 1.38 ^A^	18.76 ± 0.92 ^A^	21.52 ± 2.46 ^A^	23.29 ± 4.27 ^A^	29.24 ± 1.85 ^B^	23.26 ± 0.49 ^B^
VCL (um/s)	33.81 ± 2.83 ^A^	36.80 ± 4.40 ^A^	46.57 v 4.78 ^B^	54.76 ± 7.44 ^B^	71.62 ± 5.14 ^B^	58.68 ± 6.77 ^B^
VAP (um/s)	23.90 ± 2.00 ^A^	26.02 ± 3.10 ^A^	32.93 ± 3.39 ^B^	38.74 ± 5.26 ^B^	50.64 ± 3.64 ^B^	41.50 ± 4.80 ^B^
ALH (um)	9.90 ± 0.80 ^A^	10.78 ± 1.28 ^A^	13.64 ± 1.40 ^B^	16.04 ± 2.18 ^B^	20.98 ± 1.50 ^B^	17.19 ± 1.98 ^B^
WOB (%)	78.00 ± 6.00 ^A^	84.00 ± 2.00 ^A^	90.00 ± 2.00 ^B^	93.00 ± 2.00 ^B^	93.00 ± 4.00 ^B^	90.00 ± 5.00 ^B^
BCF (Hz)	1.19 ± 0.06 ^A^	1.00 ± 0.02	0.97 ± 0.12 ^B^	0.92 ± 0.06 ^B^	0.67 ± 0.06 ^B^	^B^ 0.66 ± 0.04 ^B^
LIN (%)	52.00 ± 6.00 ^A^	51.00 ± 4.00 ^A^	46.00 ± 2.00 ^A^	42.00 ± 2.00 ^A^	41.00 ± 5.00 ^A^	40.00 ± 4.00 ^B^
MAD (°)	70.04 ± 9.77 ^A^	84.52 ± 2.12 ^A^	86.70 ± 3.60 ^A^	110.69 ± 5.41 ^B^	35.66 ± 2.25 ^B^	39.37 ± 2.25 ^B^
STR (%)	73.00 ± 9.00 ^A^	72.00 ± 5.00 ^A^	65.00 ± 3.00 ^A^	60.00 ± 4.00 ^A^	58.00 ± 7.00 ^A^	57.00 ± 5.00 ^B^

TM, total motility; PM, progressive motility; VCL, curvilinear velocity; VSL, straight line velocity; VAP, average path velocity; ALH, mean amplitude of head lateral displacement; WOB, wobble; BCF, beat cross frequency; LIN, linearity; MAD, mean angular displacement; STR, straightness; Values are expressed as mean ± standard error of the mean; saliency analysis using the Student’s *t* test. Different labeled letters represent significant differences (*n* = 3, *p* < 0.05).

**Table 3:** The effect of pBD129 on the proliferation of *E. coli* DH5α.

	OD_450_ nm
Sperm + LB	0.363 ± 0.027 ^A^
Sperm+ *E. coli* DH5α + PBS	0.543 ± 0.047 ^B^
Sperm + *E. coli* DH5α + lgG	0.534 ± 0.119 ^B^
Sperm + *E. coli* DH5α + anti-pBD129 antibody	0.748 ± 0.060 ^C^

Different labeled letters represent significant differences (*n* = 3, *p* < 0.05).

## Supplementary Materials

The following supporting information can be downloaded at: https://www.mdpi.com/article/10.3390/ijms23169441/s1.

## Abbreviations

pBD129: porcine β-defensin 129; WHO: World Health Organization; DEFB1: human β-defensin 1; CCR6: chemokine receptor type 6; DefbΔ9: nine β-defensin genes; AR: acrosome reaction; DEFB129: β-defensin 129; LPS: lipopolysaccharide; BBD129: buffalo β-defensin 129; LB: lysogeny broth; IPTG: isopropyl-l-thiogalactopyranoside; SDS-PAGE: sodium dodecyl sulfate-polyacrylamide gel electrophoresis; LC-MS/MS: liquid chromatography/tandem mass spectrometry; MIC: minimum inhibitory concentration; PBS: phosphate buffer saline; PVDF: polyvinylidene difluoride; HRP: horseradish peroxidase; DAB: diaminobenzidine; DPBS: Dulbecco’s phosphate-buffered saline; COCs: cumulus–oocyte complexes; IVF: in vitro fertilization.

## References

[1] Barratt C.L.R., Björndahl L., De Jonge C.J., Lamb D.J., Osorio Martini F., McLachlan R., Oates R.D., van der Poel S., St John B., Sigman M. (2017). The diagnosis of male infertility: An analysis of the evidence to support the development of global WHO guidance-challenges and future research opportunities. Hum. Reprod. Update.

[2] Sharma R., Harlev A., Agarwal A., Esteves S.C. (2016). Cigarette Smoking and Semen Quality: A New Meta-analysis Examining the Effect of the 2010 World Health Organization Laboratory Methods for the Examination of Human Semen. Eur. Urol..

[3] Skakkebaek N.E., Rajpert-De Meyts E., Buck Louis G.M., Toppari J., Andersson A.M., Eisenberg M.L., Jensen T.K., Jørgensen N., Swan S.H., Sapra K.J. (2016). Male Reproductive Disorders and Fertility Trends: Influences of Environment and Genetic Susceptibility. Physiol. Rev..

[4] Krausz C., Riera-Escamilla A. (2018). Genetics of male infertility. Nat. Rev. Urol..

[5] Keck C., Gerber-Schäfer C., Clad A., Wilhelm C., Breckwoldt M. (1998). Seminal tract infections: Impact on male fertility and treatment options. Hum. Reprod. Update.

[6] Sullivan R., Légaré C., Lamontagne-Proulx J., Breton S., Soulet D. (2019). Revisiting structure/functions of the human epididymis. Andrology.

[7] Chen Y., Wang K., Zhang D., Zhao Z., Huang J., Zhou L., Feng M., Shi J., Wei H., Li L. (2020). GPx6 is involved in the in vitro induced capacitation and acrosome reaction in porcine sperm. Theriogenology.

[8] Baskaran S., Panner Selvam M.K., Agarwal A. (2020). Exosomes of male reproduction. Adv. Clin. Chem..

[9] Ni Y., Zhou Y., Chen W.Y., Zheng M., Yu J., Li C., Zhang Y., Shi Q.X. (2009). HongrES1, a cauda epididymis-specific protein, is involved in capacitation of guinea pig sperm. Mol. Reprod. Dev..

[10] Skerget S., Rosenow M.A., Petritis K., Karr T.L. (2015). Sperm Proteome Maturation in the Mouse Epididymis. PLoS ONE.

[11] Jones R. (1998). Plasma membrane structure and remodelling during sperm maturation in the epididymis. J. Reprod. Fertil. Suppl..

[12] White S.H., Wimley W.C., Selsted M.E. (1995). Structure, function, and membrane integration of defensins. Curr. Opin. Struct. Biol..

[13] Ganz T. (2003). Defensins: Antimicrobial peptides of innate immunity. Nat. Rev. Immunol..

[14] Holly M.K., Diaz K., Smith J.G. (2017). Defensins in Viral Infection and Pathogenesis. Annu. Rev. Virol..

[15] Pazgier M., Hoover D.M., Yang D., Lu W., Lubkowski J. (2006). Human beta-defensins. Cell. Mol. Life Sci..

[16] Wilson S.S., Wiens M.E., Smith J.G. (2013). Antiviral mechanisms of human defensins. J. Mol. Biol..

[17] Semple F., Dorin J.R. (2012). β-Defensins: Multifunctional modulators of infection, inflammation and more?. J. Innate. Immun..

[18] Dorin J.R., Barratt C.L. (2014). Importance of β-defensins in sperm function. Mol. Hum. Reprod..

[19] Avellar M.C.W., Ribeiro C.M., Dias-da-Silva M.R., Silva E.J.R. (2019). In search of new paradigms for epididymal health and disease: Innate immunity, inflammatory mediators, and steroid hormones. Andrology.

[20] Diao R., Fok K.L., Chen H., Yu M.K., Duan Y., Chung C.M., Li Z., Wu H., Li Z., Zhang H. (2014). Deficient human β-defensin 1 underlies male infertility associated with poor sperm motility and genital tract infection. Sci. Transl. Med..

[21] Zhou Y.S., Webb S., Lettice L., Tardif S., Kilanowski F., Tyrrell C., Macpherson H., Semple F., Tennant P., Baker T. (2013). Partial deletion of chromosome 8 β-defensin cluster confers sperm dysfunction and infertility in male mice. PLoS Genet..

[22] Tollner T.L., Bevins C.L., Cherr G.N. (2012). Multifunctional glycoprotein DEFB126--a curious story of defensin-clad spermatozoa. Nat. Rev. Urol..

[23] Sang Y., Patil A.A., Zhang G., Ross C.R., Blecha F. (2006). Bioinformatic and expression analysis of novel porcine β-defensins. Mamm. Genome.

[24] Xie K., Xie H., Su G., Chen D., Yu B., Mao X., Huang Z., Yu J., Luo J., Zheng P. (2019). β-Defensin 129 Attenuates Bacterial Endotoxin-Induced Inflammation and Intestinal Epithelial Cell Apoptosis. Front. Immunol..

[25] Xie K., Su G., Chen D., Yu B., Huang Z., Yu J., Zheng P., Luo Y., Yan H., Li H. (2021). The immunomodulatory function of the porcine β-defensin 129: Alleviate inflammatory response induced by LPS in IPEC-J2 cells. Int. J. Biol. Macromol..

[26] Dubé E., Hermo L., Chan P.T., Cyr D.G. (2008). Alterations in gene expression in the caput epididymides of nonobstructive azoospermic men. Biol. Reprod..

[27] Zhao W., Mengal K., Yuan M., Quansah E., Li P., Wu S., Xu C., Yi C., Cai X. (2019). Comparative RNA-Seq Analysis of Differentially Expressed Genes in the Epididymides of Yak and Cattleyak. Curr. Genom..

[28] Batra V., Bhushan V., Ali S.A., Sarwalia P., Pal A., Karanwal S., Solanki S., Kumaresan A., Kumar R., Datta T.K. (2021). Buffalo sperm surface proteome profiling reveals an intricate relationship between innate immunity and reproduction. BMC Genom..

[29] Xu Z., Xie Y., Zhou C., Hu Q., Gu T., Yang J., Zheng E., Huang S., Xu Z., Cai G. (2020). Expression Pattern of Seminal Plasma Extracellular Vesicle Small RNAs in Boar Semen. Front. Vet. Sci..

[30] Ren M., Cai S., Zhou T., Zhang S., Li S., Jin E., Che C., Zeng X., Zhang T., Qiao S. (2019). Isoleucine attenuates infection induced by E. coli challenge through the modulation of intestinal endogenous antimicrobial peptide expression and the inhibition of the increase in plasma endotoxin and IL-6 in weaned pigs. Food Funct..

[31] Diao H., Yu H.G., Sun F., Zhang Y.L., Tanphaichitr N. (2011). Rat recombinant β-defensin 22 is a heparin-binding protein with antimicrobial activity. Asian J. Androl..

[32] Hu Y.X., Guo J.Y., Shen L., Chen Y., Zhang Z.C., Zhang Y.L. (2002). Get effective polyclonal antisera in one month. Cell Res..

[33] Gu J., Stephenson C.G., Iadarola M.J. (1994). Recombinant proteins attached to a nickel-NTA column: Use in affinity purification of antibodies. Biotechniques.

[34] Wiegand I., Hilpert K., Hancock R.E. (2008). Agar and broth dilution methods to determine the minimal inhibitory concentration (MIC) of antimicrobial substances. Nat. Protoc..

[35] Yenugu S., Hamil K.G., Birse C.E., Ruben S.M., French F.S., Hall S.H. (2003). Antibacterial properties of the sperm-binding proteins and peptides of human epididymis 2 (HE2) family; salt sensitivity, structural dependence and their interaction with outer and cytoplasmic membranes of Escherichia coli. Biochem. J..

[36] Kawano N., Araki N., Yoshida K., Hibino T., Ohnami N., Makino M., Kanai S., Hasuwa H., Yoshida M., Miyado K. (2014). Seminal vesicle protein SVS2 is required for sperm survival in the uterus. Proc. Natl. Acad. Sci. USA.

[37] Zhang M., Su Y.Q., Sugiura K., Xia G., Eppig J.J. (2010). Granulosa cell ligand NPPC and its receptor NPR2 maintain meiotic arrest in mouse oocytes. Science.

[38] Hao Y., Mathialagan N., Walters E., Mao J., Lai L., Becker D., Li W., Critser J., Prather R.S. (2006). Osteopontin reduces polyspermy during in vitro fertilization of porcine oocytes. Biol. Reprod..

[39] Agarwal A., Baskaran S., Parekh N., Cho C.L., Henkel R., Vij S., Arafa M., Panner Selvam M.K., Shah R. (2021). Male infertility. Lancet.

[40] Zhang C., Zhou Y., Xie S., Yin Q., Tang C., Ni Z., Fei J., Zhang Y. (2018). CRISPR/Cas9-mediated genome editing reveals the synergistic effects of β-defensin family members on sperm maturation in rat epididymis. FASEB J..

[41] Yamaguchi Y., Nagase T., Makita R., Fukuhara S., Tomita T., Tominaga T., Kurihara H., Ouchi Y. (2002). Identification of multiple novel epididymis-specific beta-defensin isoforms in humans and mice. J. Immunol..

[42] Li P., Chan H.C., He B., So S.C., Chung Y.W., Shang Q., Zhang Y.D., Zhang Y.L. (2001). An antimicrobial peptide gene found in the male reproductive system of rats. Science.

[43] Yu H., Dong J., Gu Y., Liu H., Xin A., Shi H., Sun F., Zhang Y., Lin D., Diao H. (2013). The novel human β-defensin 114 regulates lipopolysaccharide (LPS)-mediated inflammation and protects sperm from motility loss. J. Biol. Chem..

[44] Zhao Y., Diao H., Ni Z., Hu S., Yu H., Zhang Y. (2011). The epididymis-specific antimicrobial peptide β-defensin 15 is required for sperm motility and male fertility in the rat (*Rattus norvegicus*). Cell. Mol. Life Sci..

