# Supplementary material for: Involvement of Porcine β-Defensin 129 in Sperm Capacitation and Rescue of Poor Sperm in Genital Tract Infection
Source: Int J Mol Sci. 2022 Aug 21;23(16):9441. doi: 10.3390/ijms23169441 (PMC9409293; doi:10.3390/ijms23169441)
Supplement: Supplementary file 1 [file ijms-23-09441-s001.zip › ijms-1858764-supplementary.pdf]

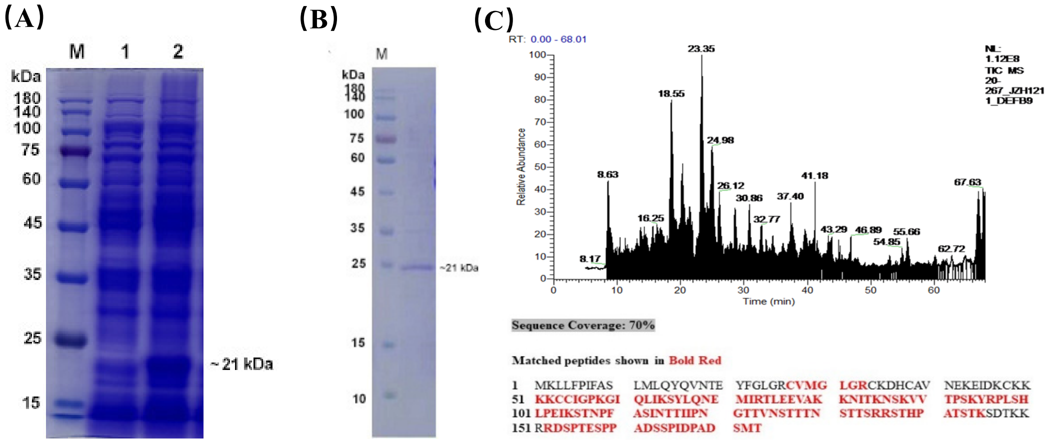

Figure S1. SDS-PAGE analysis and mass spectrometry identification of the porcine β-defensin 129. (A) M: 180 protein marker; Lane 1: *E. coli* BL21 (DE3)-pET28a(+)-pBD129 did not induced; Lane 2: *E. coli* BL21 (DE3)-pET28a(+)-pBD129 induced with IPTG. (B) M: 180 protein marker; Lane: the purification of the recombinant protein by Ni-NTA QIAexpress Kit manual. (C): Peak figure of amino acid fragments; the sequence of recombinant pBD129 protein had more than 70% match with NP_001123447 (show in red).

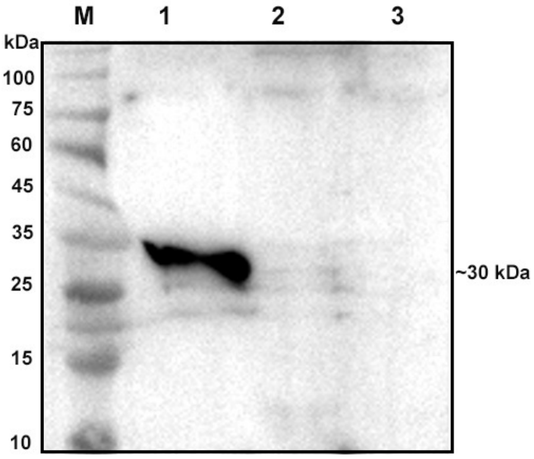

Figure S2. Western blot analysis of porcine β-defensin 129 protein expressed in HEK293T cells. M: 180 protein marker; Lane 1: Supernatant of HEK293T cells transfected with pcDNA3.1(+)-pBD129; Lane 2: Supernatant of HEK293T cells transfected with pcDNA3.1(+); Lane 3: Supernatant of HEK293T cells.

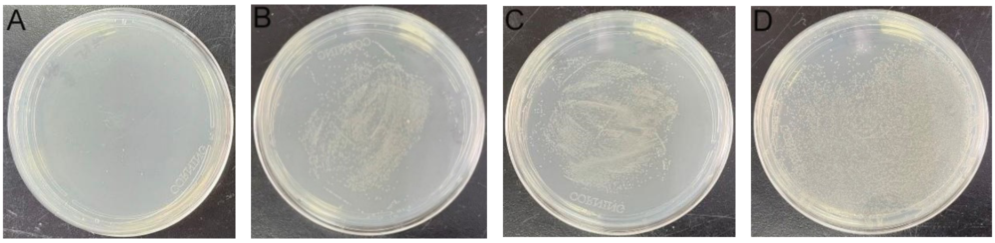

(E)

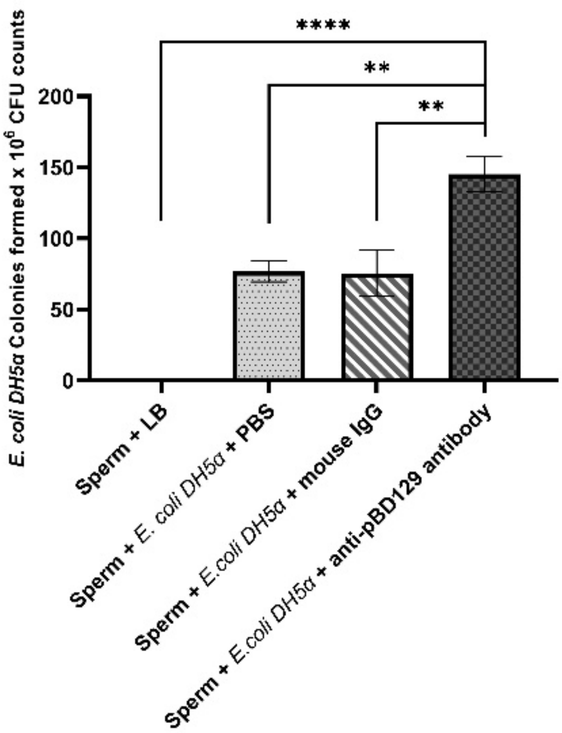

Figure S3. Representative plates from overnight bacterial cultures of E. coli after incubation with anti-pBD129 antibody or negative control for overnight. (A) Sperm + LB; (B) Sperm + *E. coli* DH5α + PBS; (C) Sperm + *E.coli* DH5α + mouse IgG; (D) Sperm + *E.coli* DH5α + anti-pBD129 antibody; (E) Summaries of colonies formed per plate (CFU). Data are presented as the mean± SEM, n=3. **$P < 0.01$; ****$P < 0.0001$.

Table S1. The effect of *E. coli* DH5α on sperm motility.

Motility parameters	Negative Control	*E. coli DH5α*-treated
TM(%)	81.27±1.92^{A}	70.76±4.14^{B}
PM (%)	61.59±4.47^{A}	53.82±4.30^{B}
VSL (um/s)	22.31±0.52^{A}	21.11±1.38^{A}
VCL (um/s)	44.40±0.84^{A}	45.58±2.78^{A}
VAP (um/s)	31.39±0.59^{A}	32.21±1.97^{A}
ALH (um)	13.00±0.25^{A}	13.35±0.82^{A}
WOB (%)	86.00±0.00^{A}	90.00±1.00^{B}
BCF (Hz)	0.79±0.03^{A}	0.80±0.05^{A}
LIN (%)	51.00±0.50^{A}	46.00±1.50^{B}
MAD (°)	116.73±6.83^{A}	101.98±13.92^{A}
STR (%)	72.00±0.50^{A}	66.00±2.40^{B}

TM, total motility; PM, progressive motility; VCL, curvilinear velocity; VSL, straight line velocity; VAP, average path velocity; ALH, mean amplitude of head lateral displacement; WOB, wobble; BCF, beat cross frequency; LIN, linearity; MAD, mean angular displacement; STR, straightness; Values are expressed as mean ± standard error of the mean; saliency analysis using the *Student's t* test. Different labeled letters represent significant differences (n=3, $P < 0.05$).
